# Supplementary material for: Structures of Three Actinobacteriophage Capsids: Roles of Symmetry and Accessory Proteins
Source: Viruses. 2020 Mar 8;12(3):294. doi: 10.3390/v12030294 (PMC7150772; doi:10.3390/v12030294)
Supplement: Supplementary file 1 [file viruses-12-00294-s001.pdf]

Supporting information

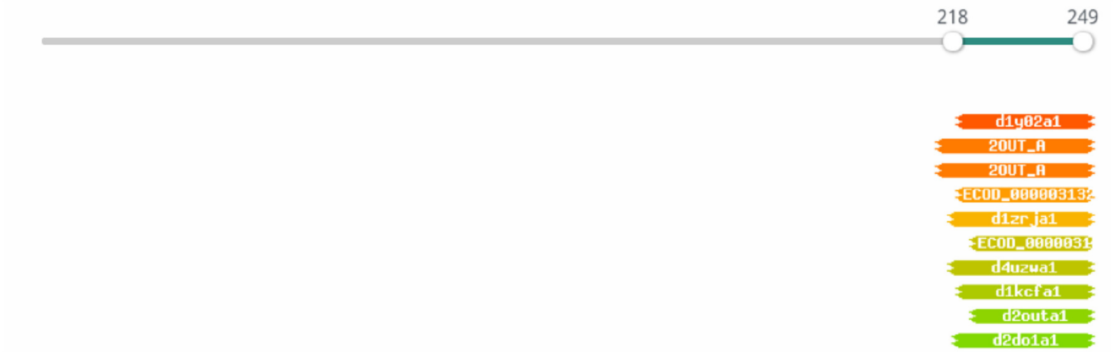

Hit	Name	Probability	E-value	SS	Cols
d1y02a1	Rififylin (FYVE-RING finger protein Sakura) {Homo spaiens}	92.56	0.36	4	32
2OUT_A	Mu-like prophage FluMu protein gp35 {Haemophilus influenzae}	89.61	1.1	4	37
ECOD_000003132_e1y02A1	Domain A:alpha arrays, X:"LEM/SAP HeH motif", F: KOG4275	86.75	3.3	4	32
d1zrja1	Heterogenous nuclear ribonucleoprotein U-like protein 1 {Homo spaiens}	84.59	4.3	3.3	32
ECOD_000003126_e2outA1	Domain A:alpha arrays, X:"LEM/SAP HeH motif-like", T:"LEM/SAP HeH motif", F:HeH	81.93	6.9	3.6	29
d4uzwa1	Automated matches (A:2-50) {Saccharomyces cerevisiae}	81.16	7	3.3	32
d1kcfa1	Mitochondrial resolvase ydc2 N-terminal domain {Schizosaccharomyces pombe}	80.19	10	3.8	30
d2outa1	Uncharacterized protein HI1507 in Mu-like prophage FluMu region {Haemophilus influenzae}	78.35	11	3.6	29
d2do1a1	Nuclear protein hcc-1 {Homo spaiens}	77.62	13	3.4	31

Supporting Figure S1. HHPRED prediction of Rosebush gp17. The amino acid sequence of gp17 was used as the input into HHPRED and searched against all the available databases.

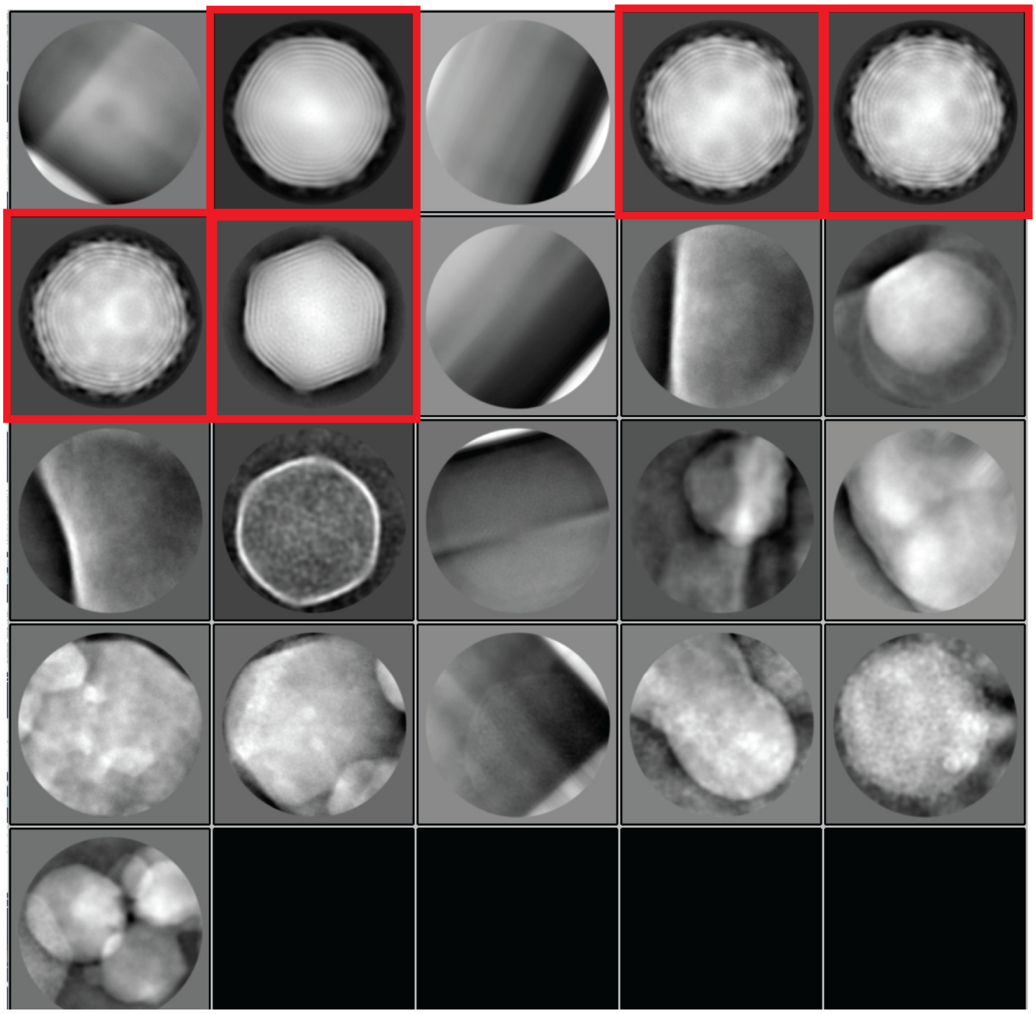

Supporting Figure S2. 2D classes selected from multiplexed Rosebush and Patience cryo-EM grid. The red boxes highlight the “good” classes chosen for 3D classification and refinement.

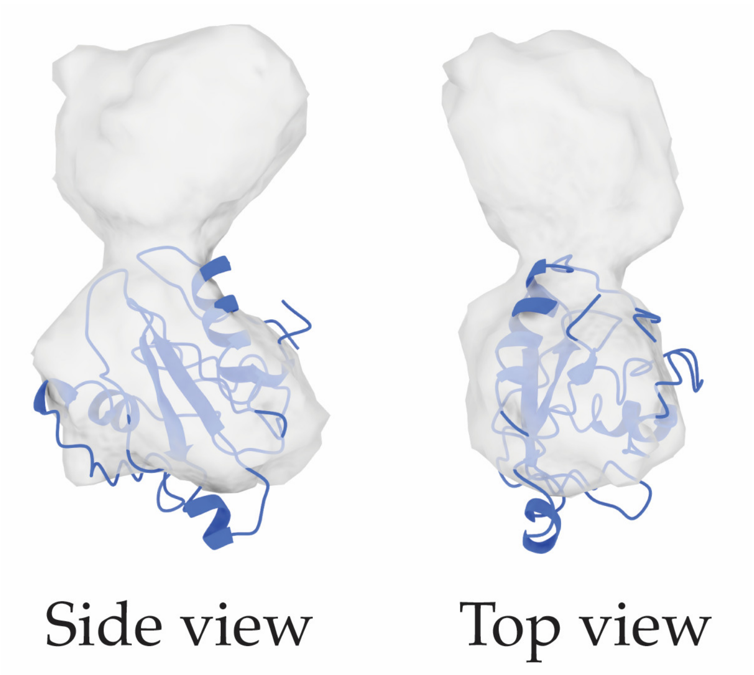

Supporting Figure S3. I-TASSER predicted model of Patience gp29 fitted into decoration protein density using ChimeraX.

```
Pupper            -MLRVPVDNPTLRRTLRPLYAQHQATPWGGFLDPTYDPATDPEIYPGTVMKRADLGTARD        59
E3                -MFKTPIQNATQKRTIRPQYAQHQATAWAGWLDPAWDRK--FDVLPGMVMCRKG------        51
Myrna             -MFRPPLANPAQKRTLRPLYANHQATPWGGFLDPDLDVD--FDIYPGTVMQRV-------        50
Alice             MTFRPPASNPGQKRTLVPRYANTQATPWAGFLDPDLDVD--FDILPGTVMQRL-------        51
Phrappuccino      -MFRVTPANPAQKRTIRPVYAQHQATAYAGFLDPAWDRS--FDIVPGTVMSRK-------        50
Stormageddon      -MFMVPVSNPAIKRTIRPLYAQHQATTWAGFLDPNWDRS--FDILPGCVMTRL-------        50
Finch             -MFRVPLANPSLKRLLRPLFAQHQATPKPGLLDPTWDRS--FDIYPGSVMTRL-------        50
                    :     *   :* : * :*: ***   * ***  *     :: ** ** *

Pupper            GGEVWKPYDGT---GEPVGLSALFMAP----SMGVNEVTASGTNLFTIWVGGSDAVFEIL        112
E3                PGEVFAPFESVAASGKGFGLAALFLAP----KMQIDEVTPTGTNAFTVWVGNNNAAFEIL        107
Myrna             YGEVFAPYTGEA-GTSPVGLSALFVNTGGYQGHQVNEVTGSGTGLFTIWIGGPDAMFEIL        109
Alice             YGDVFAPYTGEA-GTVPFGLSALFHAP----RLGVTEVSSAGTGLFTVWVGDSQAVFDVL        106
Phrappuccino      SGEVFEPFTGKP-GQKPFGLSALFAAP----RLGIDEVTGTGANNFTVWVGGEQAVFEIL        105
Stormageddon      SKEVFAPYTGQA-NAKPFGLSAFFMAP----RLGVEEVTGTGQNLFTVWVGNDDAEFEIL        105
Finch             FGDVYAPYTGAA-GQKPFGLSAFFVAP----KLGVDEVLDTGSNLFTVWVGNNDAEFEVL        105
                    :*: *: .       .**:*:*          : **  :* . **:*:*. :* *::*

Pupper            SPAFDPTGLNTAE------AGAYLTGNSDGLLTVT---GATNANSIAQLVEVVSDAKIIV        163
E3                APAFDQTATWTEPGPGRVLLFPSLVAGQIGKLTPTKGAGMDDTDAVAELLDVIDANTILV        167
Myrna             APAFDTEADWPEV--TGPG-KYMLTANAQGQLTPE---GVNPGNAIATLIDIPSTDKIII        163
Alice             APAFDVEADWPAAGETGPA-RVMLTANSKGRLTPD---GVTPENVIAELIDIPSTDKITI        162
Phrappuccino      NPGFDLDADWNAANVTDGS-FKLLTATDTGLLTPT---GVDHGNAVAELIDVISTDAIQI        161
Stormageddon      APAFDTTADWTLP--TNGS-IKLLTGNSQAKLTPA---GATVYNAIAELIDVVGTDKIVI        159
Finch             APAFDATADWTTP--TNGS-IVLLTGNNKGLLTPT---GATAANAIAELLSVNGTEKITI        159
                   *.**  .               *..   . **     *    : :* *:.: .   * :

Pupper            RLHRKVTA----------  171
E3                SLNRQK------------  173
Myrna             SLDRYDIAASGGGLAGGS  181
Alice             RLNRLDLAATGGGLAGGS  180
Phrappuccino      RLNRYDLT-SAVALAGGS  178
Stormageddon      RPFRPTPG-TGA------  170
Finch             RPVRPTA-----------  166
                     *
```

Supporting Figure S4. Multiple sequence alignment of bacteriophage proteins that are related (in the same pham) to Myrna minor coat protein 98. Alignment was done using Clustal Omega. Information of each bacteriophage aligned is as follows. **Pupper**: DO subcluster, gene 95, accession number MK977695. **E3**: Singleton, gene 81, no accession number. **Myrna**: C2 subcluster, gene 98, accession number EU826466. **Alice**: C1 subcluster, gene 91, accession number JF704092. **Phrappuccino**: AA subcluster, gene 54, accession number MK937592. **Stormageddon**: DX subcluster, gene 14, accession number MN586040. **Finch**: Singleton, gene 18, accession number MG962366.

```
Rosebush             MPAGIAVE-LDNGFATLEFLDKAARGRALEALIRVGGPATIEKLTREKGARSVRYRVPEG      59
Arbiter              MPAGIAVE-LDNGFATLEFLDKAARGRALEALIRVGGPATIEKLTREKGARSVRYRVPEG      59
Hosp                 MPAGIIAT-IDGDYATIDFVNQALRGPALAELAELGAP--IETITRD--GPRRKYRVLVN      55
Thonko               MPEGIIAT-VEGGFATVDIIDPQTRHEKLAELIEIGGPGSVETITRV--GPRRQYRVPVG      57
Phelemich            MPAGIIAT-VDDGYATVDFVDPTLRGPGLQAVIDIAGPHAIETITRK--GPRRQYKMLVG      57
LilMcDreamy          MPEGIVAT-VEDGYATLDFVDVTKRGPALQALIELAGPDSIETITRR--GPRRQYRVLVD      57
Athena               MPAGIEVV-VEDGFATIDFLDPKLKGPALGRLIASGGPGVVEPLTREKGSPRKRYRVPEG      59
TPA2                 MPAGIEVE-VDNGFATLDFVNKSLRGPALAKLIEQGGGEIVETITRE--GPRRKYRVPLG      57
BirdsNest            MPEGIIATDHGDGFVTLDFVDKSLRGPALDELVKLGGAETIETISRR--GPRRQYKVPLG      58
Apex                 MPAGIIAT-VDNGFATIDFVDKSLRGPALAELVEIGGPASIETITRD--GPRRKYRVPAG      57
Imvubu               MPEGIIATDHGDGFVTLDFVDKALRGPALAELVEIGGAASIETISRV--GPRRQYRVPLG      58
Indlovu              MPEGIVAT-VEDGYATLDFVDPTLRGAALTKLLDIGGPGTIETLTRV--GPRRQYRVPEG      57
Saguaro              MPQGIIAT-VDDGFATIDFVDPSLRGPALTRLVEIGGPETIETLTRV--GPRRQYRVPEG      57
Quesadilla           MPEGILAT-VDDGFATIDFVDPALRGPALAQLVELGGASSIETITRV--GPRRQYRVPEG      57
Anderson             MPEGIIAT-VEDGFATIDFVNPDLRGPALAALLELGGPGTIETISRR--GPRRLYRVPEG      57
AbsoluteMadLad       MPEGIIAT-VEDGFATIDFVNPELRGPALAALLELGGPGTIETISRR--GPRRLYRVPEG      57
CloverMinnie         MPEGIIAE-VEDGFATIDFVDRSLRGPGLSKLLEFG-PETVETLTRS--GPRRVYRVPEG      56
                     ** ** .    ..:.*:::::   :   *  :   .    :* ::*   .    *::  .

Rosebush             NAREAGLLDDYEVGLSNV--------------------------VASGGTDATG-----I      88
Arbiter              NAREAGLLDDYEGGLSNV--------------------------VASGGTDATG-----I      88
Hosp                 FAEQANLLDLDENGDPTAPG-----------------------AVFSAGHDTGAAAALVA      92
Thonko               NAQAAGLLDGDEVAA-----------------------------VRTAGHDSGAADALVA      88
Phelemich            NAEAAGLLDGDESGR-----------------------------LRTAGPDSGTAAALVA      88
LilMcDreamy          NADAVGLLDGDEVAR-----------------------------PRSAGNDSGARDALVA      88
Athena               NAREAGLLDEAREVDALVSN------------------------VLESGVDAHG------      89
TPA2                 NAQAAGLIDGSTPVTP----------------------------VKSAGRDSGSAAALKA      89
BirdsNest            NAVELGLVDDAAELAPRGAGAN----------------EDGTARGASAGADTGAAARTKA      102
Apex                 NAQAAGLLDGD-EVG----------------------------DVWSAGRDTGAAAATKA      88
Imvubu               NATEAGLVDDENTVE----------------------------GVRSAGQDTGRAAALKA      90
Indlovu              NAREAGLLDDATEEHPARGSTAFADSDPENDALTSEGGVRGAFTPGSAGTDTGAAAALKA      117
Saguaro              NAREAGLLDDETGEHPRGAV----QYSAEDAALESEGGVRGAFTPGNAGTDTGAAAALAA      113
Quesadilla           NAREVGLLDEATGEHARGAV----DHSEEDAALDPEGGVRGAFTPGNAGVDTGAAAALKA      113
Anderson             NAEEVGLLDGDEGPAKW---------------------------SAGA--DTGRAAALKA      88
AbsoluteMadLad       NAEEVGLLDGDEGPATW---------------------------SAGA--DTGRAAALKA      88
CloverMinnie         NAREVGLLDAASEADDD---------------------------KGQDDKDSGAGDDT--      87
                      *   .*:*                                         *:

Rosebush             VLPETPTSKGL-----VHG---QQLRSGTYRGGDTGNVHTTTPSDPV----------ARG      130
Arbiter              VLPETPTSKGL-----VHG---QQMRSGTYRGGDTGNVHTTTPSDPV----------ARG      130
Hosp                 ADPNVN--EGA-DKANWHTPVAEYTSANKFVGQVPN-SQVLD-RVQVHTGDASSYGGS--      145
Thonko               ADPNVN--AGT-DAAEWHTPTAEHTSRNAYVGQTSA-ADERAMAPSPFTGKGTSYGGGNA      144
Phelemich            ADPNVN--AGA-DNADWHTPYDEYTSANKYVGATTV-TAARAAADPAFTGTATPHGGTNA      144
LilMcDreamy          ADPNVN--PGE-DNANWHQQTTEVGTRNTYVGATTS-AEERAMTPPRFTGTATPFGGSSA      144
Athena               SAPDLP------------------TSANAHSQT---------TRS---GSYSAAYGGENN      119
TPA2                 AKSTGP--------------YPFPTSRNKWSTGDPA-ATADLLGFQALTAQGV-----DL      129
BirdsNest            SDPNVN--KGS-DQADWHTPVAEHTSANAYVGQVPN-AQVLHGRPQVFTGDADSYGGRSA      158
Apex                 SDPNVN--PGA-DNANWHTPVDQYTSANKYVGQVPN-ATVLHNRGQVYTGDADSVGGDLE      144
Imvubu               ADPNVN--PGA-DNADWHTPVAEHTSANAYVGQVPN-SQVL-DRAQVFTGDAGSYGGSGK      145
Indlovu              ANPNVN--AGA-DGADWHTPTAEYTSANAYVGETTA-AEERAMAPSPFTGLDAPVGGKNA      173
Saguaro              ADPNTN--PGT-DNANWHTPVAEHTSANKYVGETTV-TEARAAADPAYTGTASSYGGKNA      169
Quesadilla           ANPNTN--ATPANAGDWHTPTAEYTSENKYVGTTSA-AEERALAPSPFTGSATSHGGSNA      170
Anderson             ADPNVV------GGDDWHTPILEHSSRNAYVGTVAN--EDVLDRTQVYTGSAHSFGGHAV      140
AbsoluteMadLad       ADPNVV------GGDDWHTPILEHSSRNAYVGTVAN--EDVLDRTQVYTGSAHSFGGHAV      140
CloverMinnie         ------------------------------------------------------------      87


Rosebush             IATPTHAEVIEHVAASGPVQPQGVPA----------------------------------      156
Arbiter              IATPTHAEVIEHVAASGPVQPQGVPA----------------------------------      156
Hosp                 GQAPTHREVIEHVKMRGGTL--QAVQ------------TMSFGHPGAEANLSLADQPSAL      191
Thonko               SEVPTHRDVIEHVKQARVDNPVPVGT------------AGPAERGAVKANLGLAEQTSAI      192
Phelemich            GDTPTHAEVIAHVKKAHPEGGFVG------------------------------------      168
LilMcDreamy          ADTPTHAEVIEHVKGHRVTPSQWA-D------------PTDLEHSGAAANLRLASIQSVE      191
Athena               PTPEVPAEALSTVSVA-------------------------------------V-------      136
TPA2                 PD---STWKLDEMRTFAREQEI------------DTTGLTTKAATLERIEDALAAR----      170
BirdsNest            PENETHRELIDRVKEGSSVLAVGGV-------QPATEVEPRALTPVSQINASLKSQTGAL      211
Apex                 -HPPTHADIIQHVKDA--------K-------TPPTEGFAAPMARAAVVAGSLAEQDAAL      188
Imvubu               --ATTHRDLIEHVKANSSVLAVGGV-------QPATEGFAPMAMRASEISGALADQTAAL      196
```

```
Indlovu             AETQTHREVIDQVTEYKHANPK-------GHARGAEAQTAVRQAPAQHLNTALKDQAGAL      226
Saguaro             AESESHRQLIDRVKAHELANPK-------GAPRGEAA----PHAPVQSLNTGLVAQTGAL      218
Quesadilla          AETPTHREVIDRVKEFKQANPK-------GAPRGEAA----PHAPVQALNTGLKDRDSAL      219
Anderson            APT--STDLLDGLDAIKYPQPPEEVPPGEGEG----DGEGIEGMSAQRVNLGLAEQTSGL      194
AbsoluteMadLad      APT--STDLLDGLDAIKYPQPPEEVPPGEGEGDGEGDGEGIEGMSAQRVNLGLAEQTSGL      198
CloverMinnie        ------------------------------------------------------------      87

Rosebush            --EAYPAPEPGGAYIGMQGLGEA-----VTPAE-AETSGSGD------------------      190
Arbiter             --EAYPAPEPGGAYIGMQGLGED-----VTPAE-AETSGSGD------------------      190
Hosp                ATDPGSTPDVGGSFVAEDYTSVQ-ATRDAPLAGPEDVTVIGPDGE----------PENTE      240
Thonko              GTDPQARVVDT--------------TEAEPVSA---------------------------      211
Phelemich           -STPGG------------------------------------------------------      173
LilMcDreamy         STDPQGATILPGDKVALA-------TTEEPVDD----------GE----------TDTGA      224
Athena              G-AAQPWD------------EPA-------------------------------------      146
TPA2                --DPEP-----EVVQV---ETPADDTATEAPADTETAE----------------------      198
BirdsNest           GDDPGARPDAGGEALGD-YSTPQ-SQRVESPQDGQTTETLGHVSSVPSGSE-----RVQT      264
Apex                GSDPGGWGPQPGEAAPEAPSEPQ-------------------------------------      211
Imvubu              GSDPGAWGEPGGEALAEDYTSVQ-ATREGESAQGEDQTGDAVITNDPGVSDSPTPPTIVE      255
Indlovu             ASDPGGWAEQPDADHPSVGLAPA-A------------EGSE------DIQT---------      258
Saguaro             ADDPGARPDEGGQARST-YSTVQ-STRQAPRAKEQRKSGT----------A---------      257
Quesadilla          ADDPGARPDEGGAVRGTTYSTPQ-SQRAEGAQISQEARGGSRAAEVPGAAD---------      269
Anderson            ASDPGATPEVGGEALGEF-TTV--------------------------------------      215
AbsoluteMadLad      ASDPGATPEVGGEALGEF-TTV--------------------------------------      219
CloverMinnie        ------------------------------------------------------------      87

Rosebush            ---------------GGVVVDVPGDDDIVAKQAWPAGEPNEDWKRSELDAYAAS-IGINT      234
Arbiter             ---------------GGVVVDVPGDDDIVAKQAWPAGEPNEDWKRSELDAYAAS-IGINT      234
Hosp                NASQPPAEPVTEGTQAEPVTEAD---GEPRPDGLPEGEPNADWLRPQLEAYATW-KGIE-      295
Thonko              ------------------TPRGF---TASEDGAQGEEAPTEKWTRKDIDQWAAK-RGIDT      249
Phelemich           ------------------------------EPIYPDGAPSLEWTRAQLDAYAAA-MTPPI      202
LilMcDreamy         GASEPL---------PVETTTGD---RGPRPDGLPEGEPNADWLRPQLETYATW-IGLD-      270
Athena              ----------------------VPQELPVAEKAYPEGEPDDEWTRPELNAYATD-HGI--      181
TPA2                ------------------------------------------------------------      198
BirdsNest           EASPGTTEPS---SEPEPTNEPVSKPDTEDVVEYPEGEPTKEWRRDELDAYALKVKGLDT      321
Apex                -------------SDETPAAEVT---TPADAPAYPDGEPTVDWTRKQLDAYAADKLDLDT      255
Imvubu              GGDPTTGDPGTQGGDEPTTLEPT---PAPATQPYPEGTPTEDWKRAELDAYALAVKGIDT      312
Indlovu             -------------QASAGTAEGITAPDVEDVVEYPQGEPTSDWRRDELDAYALKVKGLDT      305
Saguaro             -------------AKSEVIAPPVTAPDTEDVVEYPEGDPSTSWRRDELDAYALKVKGLDT      304
Quesadilla          -------------SGSQEAAGGTTPPDTEDVVEYPEGEPSDKWRRDELDAYALKVKSLDT      316
Anderson            -----------------------QSTRVEDAAAYPEGEPSEDWKRAELDAYAAA-HGLDT      251
AbsoluteMadLad      -----------------------QSTRVEDAAAYPEGEPSEDWKRAELDAYAAA-HGLDT      255
CloverMinnie        ------------------------------PTDYPDGEPDSEWKFDQLKAYAAD-HEVDI      116

Rosebush            KNL---DNKAAVLDALKK--------  249
Arbiter             KNL---DNKAAVLDALKK--------  249
Hosp                N-PSGYPNKAELLTAIENA-------  313
Thonko              T-DAGT--KAEALDIIAKAQKG----  268
Phelemich           D-TTGLANKQAVLDAIAANQ------  221
LilMcDreamy         N-PSGYPNKTELLTAIENKEQS----  291
Athena              EGAANFPNKAALLKAIKDAE------  201
TPA2                --------------------------  198
BirdsNest           TSL---GNKTEVLDAIKNAPKA----  340
Apex                TKL---ESKAAVVAAINAPKE-----  273
Imvubu              TQE---PNKAAVVAAIQNAPTPT---  332
Indlovu             SSKSDFPAKKDVLAAINSAK------  325
Saguaro             SNRSDFPSKDAVLDAIQQKG------  324
Quesadilla          SGL---GSKAEVLKAIQKGK------  333
Anderson            TKL---ANKGEVLAAINEAADGEDDA  274
AbsoluteMadLad      TKL---ANKGEVLAAINEAADGEDDA  278
CloverMinnie        DGA---RSKADVLAKIKEQSQ-----  134
```

Supporting Figure S5. Multiple sequence alignment of bacteriophage proteins that are related (in the same pham) to Rosebush gp17. Alignment was done using Clustal Omega. Information of each bacteriophage aligned is as follows. **Rosebush**: B2

subcluster, gp17, accession number AY129334. **Arbiter**: B2 subcluster, gp17, accession number JN618996. **Athena**: B3 subcluster, gp17, accession number JN699003. **Apex**: B4 subcluster, gp14, accession number MN428050. **Phelemich**: B5 subcluster, gp15, accession number KF416341. **Hosp**: B6 subcluster, gp10, accession number KJ433974. **Saguaro**: B7 subcluster, gp16, accession number MH744423. **Thonko**: B8 subcluster, gp15, accession number MH632120. **Quesadilla**: B9 subcluster, gp16, accession number MN617843. **Imvubu**: B10 subcluster, gp11, accession number MN813693. **Indlovu**: B11 subcluster, gp14, no accession number. **LilMcDreamy**: B12 subcluster, gp15, accession number MN284893. **BirdsNest**: B13 subcluster, gp19, accession number MN813686. **TPA2**: Singleton, gp23, accession number HM486077. **Anderson**: UNK subcluster, gp14, no accession number. **CloverMinnie**: DR subcluster, gp15, accession number MN234196.

Table S1. Mass spectrometry data analysis of band 1 from SDS-PAGE of Rosebush capsid

Protein name	Protein accession numbers	Total spectrum count	Percentage of total spectra	Percentage sequence coverage
Uncharacterized protein Mycobacterium phage Rosebush Gp=34	Q857A0_9CAUD	3	0.47%	25.60%
Uncharacterized protein Mycobacterium phage Rosebush Gp=38	Q856Z6_9CAUD	10	1.57%	13.30%
Uncharacterized protein Mycobacterium phage Rosebush Gp=49	Q856Y5_9CAUD	6	0.94%	13.00%
WW domain-containing protein Mycobacterium phage Rosebush Gp=32	Q857A2_9CAUD	9	1.42%	12.10%
Uncharacterized protein Mycobacterium phage Rosebush Gp=53	Q856Y1_9CAUD	3	0.47%	9.82%
Major tail protein Mycobacterium phage Rosebush Gp=21	Q857B3_9CAUD	3	0.47%	9.19%
Uncharacterized protein Mycobacterium phage Rosebush Gp=62	Q856X2_9CAUD	2	0.31%	7.30%
Uncharacterized protein Mycobacterium phage Rosebush Gp=15	Q857B9_9CAUD	3	0.47%	5.93%
Head-to-tail adaptor Mycobacterium phage Rosebush Gp=24	Q857B0_9CAUD	1	0.16%	5.85%
Uncharacterized protein Mycobacterium phage Rosebush Gp=48	Q856Y6_9CAUD	1	0.16%	5.59%
Uncharacterized protein Mycobacterium phage Rosebush Gp=2	Q857D2_9CAUD	2	0.31%	4.68%
Uncharacterized protein Mycobacterium phage Rosebush Gp=37	Q856Z7_9CAUD	1	0.16%	4.02%
Uncharacterized protein Mycobacterium phage Rosebush Gp=31	Q857A3_9CAUD	1	0.16%	3.44%
Uncharacterized protein Mycobacterium phage Rosebush Gp=45	Q856Y9_9CAUD	1	0.16%	3.15%
Tape measure protein Mycobacterium phage Rosebush Gp=29	Q857A5_9CAUD	6	0.94%	2.87%
POLAc domain-containing protein Mycobacterium phage Rosebush Gp=56	Q856X8_9CAUD	1	0.16%	1.81%
Capsid maturation protease and MuF-like fusion protein Mycobacterium phage Rosebush Gp=13	Q857C1_9CAUD	2	0.31%	1.47%
Bovine serum albumin precursor	CON__P02769	426	67.00%	0.00%
ALB isoform 1 of serum albumin precursor	CON__P02768-1	70	11.00%	0.00%
KRT1 Keratin, type II cytoskeletal 1	CON__P04264	42	6.60%	0.00%
KRT10 Keratin, type I cytoskeletal 10	CON__P13645	32	5.03%	0.00%
Keratin, type II cytoskeletal 2 epidermal	CON__P35908	19	2.99%	0.00%
Keratin, type I cytoskeletal 9	CON__P35527	15	2.36%	0.00%
Isoform 1 of Keratin, type I cytoskeletal 10	CON__P02535-1	14	2.20%	0.00%
TRYP_PIG Trypsin (Sus scrofa)	CON__P00761	13	2.04%	0.00%
KRT14 Keratin, type I cytoskeletal 14	CON__P02533	9	1.42%	0.00%
KRT16 Keratin, type I cytoskeletal 16	CON__P08779	9	1.42%	0.00%
HRNR Hornerin	CON__Q86YZ3	7	1.10%	0.00%
KRT2 Keratin, type II cytoskeletal 2 epidermal	CON__Q3TTY5	6	0.94%	0.00%
Krt13 Isoform 1 of keratin, type I cytoskeletal 13	CON__P08730-1	5	0.79%	0.00%
Keratin, type II cytoskeletal 6C	CON__P48668	4	0.63%	0.00%

KRT5 Keratin, type II cytoskeletal 5	CON__P13647	4	0.63%	0.00%
KRT6A Keratin, type II cytoskeletal 6A	CON__P02538	4	0.63%	0.00%
Kertain, type I cuticular HA2 (Hair keratin, type I HA2)	CON__Q14532	3	0.47%	0.00%
KRT73 Keratin-73	CON__Q32MB2	3	0.47%	0.00%
KRT77 Keratin 77	CON__Q7Z794	3	0.47%	0.00%
CYTOKERATIN homolog	CON__Q8BGZ7	3	0.47%	0.00%
Apolipoprotein E precursor (Bos taurus)	CON__Q03247	2	0.31%	0.00%
Vitamin D-binding protein precursor (Bos taurus)	CON__Q3MHN5	2	0.31%	0.00%
Similar to gelsolin (Bos taurus)	CON__Q3SX14	2	0.31%	0.00%
Tropomyosin 2 (Bos taurus)	CON__Q3SX28	1	0.16%	0.00%
Beta-casein (Bos taurus)	CON__P02666	1	0.16%	0.00%
Streptavidin	CON__Streptavidin	1	0.16%	0.00%
Similar to collagen aplha 1(III) chain (Bos taurus)	CON__P04258	1	0.16%	0.00%
Alpha-1-antiproteinase precursor (Bos taurus)	CON__P34955	1	0.16%	0.00%
Keratin, type I cuticular HA4 (Hair keratin, type I HA4)	CON__O76011	1	0.16%	0.00%
Similar to complement C4-A precursor (Bos taurus)	CON__ENSEMBL:ENSBTAP00000007350	1	0.16%	0.00%
54 kDa protein (Bos taurus)	CON__ENSEMBL:ENSBTAP00000018229	1	0.16%	0.00%
Similar to inter-alpha-trypsin inhibitor heavy chain 2 (Bos taurus)	CON__Q9TRI1	1	0.16%	0.00%

Table S2. Mass spectrometry data analysis of band 2 from SDS-PAGE of Rosebush capsid

Protein name	Protein accession numbers	Total spectrum count	Percentage of total spectra	Percentage sequence coverage
Uncharacterized protein Mycobacterium phage Rosebush Gp=34	Q857A0_9CAUD	5	0.35%	18.60%
Uncharacterized protein Mycobacterium phage Rosebush Gp=37	Q856Z7_9CAUD	3	0.21%	17.10%
Uncharacterized protein Mycobacterium phage Rosebush Gp=38	Q856Z6_9CAUD	7	0.50%	14.00%
Uncharacterized protein Mycobacterium phage Rosebush Gp=40	Q856Z4_9CAUD	1	0.07%	13.20%
Uncharacterized protein Mycobacterium phage Rosebush Gp=17	Q857B7_9CAUD	2	0.14%	10.00%
Uncharacterized protein Mycobacterium phage Rosebush Gp=39	Q856Z5_9CAUD	1	0.07%	9.32%
Uncharacterized protein Mycobacterium phage Rosebush Gp=31	Q857A3_9CAUD	2	0.14%	7.16%
Uncharacterized protein Mycobacterium phage Rosebush Gp=53	Q856Y1_9CAUD	1	0.07%	6.75%
Uncharacterized protein Mycobacterium phage Rosebush Gp=45	Q856Y9_9CAUD	1	0.07%	4.72%
Uncharacterized protein Mycobacterium phage Rosebush Gp=48	Q856Y6_9CAUD	1	0.07%	4.47%
Major tail protein Mycobacterium phage Rosebush Gp=21	Q857B3_9CAUD	1	0.07%	4.18%
Tape measure protein Mycobacterium phage Rosebush Gp=29	Q857A5_9CAUD	7	0.50%	4.14%

Capsid maturation protease and MuF-like fusion protein Mycobacterium phage Rosebush Gp=13	Q857C1_9CAUD	3	0.21%	3.68%
Bovine serum albumin precursor	CON__P02769	1069	75.50%	0.00%
ALB isoform 1 of serum albumin precursor	CON__P02768-1	110	7.77%	0.00%
KRT10 Keratin, type I cytoskeletal 10	CON__P13645	80	5.65%	0.00%
KRT1 Keratin, type II cytoskeletal 1	CON__P04264	54	3.82%	0.00%
KRT2 Keratin, type II cytoskeletal 2 epidermal	CON__P35908	36	2.54%	0.00%
TRYP_PIG Trypsin (Sus scrofa)	CON__P00761	34	2.40%	0.00%
KRT5 Keratin, type II cytoskeletal 5	CON__P13647	25	1.77%	0.00%
KRT14 Keratin, type I cytoskeletal 14	CON__P02533	20	1.41%	0.00%
Krt10 isoform 1 of keratin, type I cytoskeletal 10	CON__P02535-1	19	1.34%	0.00%
KRT16 Keratin, type I cytoskeletal 16	CON__P08779	17	1.20%	0.00%
KRT6A Keratin, type II cytoskeletal 6A	CON__P02538	15	1.06%	0.00%
KRT9 Keratin, type I cytoskeletal 9	CON__P35527	14	0.99%	0.00%
KRT6C Keratin, type II cytoskeletal 6C	CON__P48668	14	0.99%	0.00%
KRT3 Keratin, type II cytoskeletal 3	CON__P12035	11	0.78%	0.00%
54 kDa protein (Bos taurus)	CON__ENSEMBL:ENSBTAP00000018229	11	0.78%	0.00%
63 kDa protein (Bos taurus)	CON__ENSEMBL:ENSBTAP00000038253	11	0.78%	0.00%
Vitamin D-binding protein precursor (Bos taurus)	CON__Q3MHN5	11	0.78%	0.00%
Krt13 Isoform 1 of keratin, type I cytoskeletal 13	CON__P08730-1	10	0.71%	0.00%
SERPINA3-4 (Bos taurus)	CON__A2I7N0	8	0.57%	0.00%
KRT2 Keratin, type II cytoskeletal 2 epidermal	CON__Q3TTY5	7	0.50%	0.00%
Similar to endopin 2B (Bos taurus)	CON__REFSEQ:XP_001252647	6	0.42%	0.00%
KRT77 Keratin 77	CON__Q7Z794	6	0.42%	0.00%
Keratin, tyoe I cuticular HA2 (Hair keratin, type I HA2)	CON__Q14532	5	0.35%	0.00%
KRT73 Keratin-73	CON__Q6NXH9	5	0.35%	0.00%
HRNR Hornerin	CON__Q86YZ3	3	0.21%	0.00%
FLG2 Filaggrin-2	CON__Q5D862	3	0.21%	0.00%
Alpha-1-antiproteinase precursor (Bos taurus)	CON__P34955	3	0.21%	0.00%
Tropomyosin 2 (Bos taurus)	CON__Q3SX28	2	0.14%	0.00%
Alpha-1-acid glycoprotein precursor (Bos taurus)	CON__Q3SZR3	2	0.14%	0.00%
Beta-casein (Bos taurus)	CON__P02666	2	0.14%	0.00%
9 kDa protein (Bos taurus)	CON__ENSEMBL: ENSBTAP00000038329	2	0.14%	0.00%
Similar to inter-alpha-trypsin inhibitor heavy chain 2 (Bos taurus)	CON__Q9TRI1	1	0.07%	0.00%
LUXY_VIBFI Yellow fluorescent protein (YFP) Vibrio fischeri	CON__P21578	1	0.07%	0.00%
Amylase, alpha 2B; panreatic (Bos taurus)	CON__Q3MHH8	1	0.07%	0.00%

Table S3. Mass spectrometry data analysis of band 3 from SDS-PAGE of Rosebush capsid

Protein name	Protein accession numbers	Total spectrum count	Percentage of total spectra	Percentage sequence coverage
Uncharacterized protein Mycobacterium phage Rosebush Gp=34	Q857A0_9CAUD	4	0.70%	31.00%
Uncharacterized protein Mycobacterium phage Rosebush Gp=17	Q857B7_9CAUD	11	1.93%	23.30%
Uncharacterized protein Mycobacterium phage Rosebush Gp=42	Q856Z2_9CAUD	2	0.35%	13.90%
Capsid maturation protease and MuF-like fusion protein Mycobacterium phage Rosebush Gp=13	Q857C1_9CAUD	17	2.98%	11.40%
Uncharacterized protein Mycobacterium phage Rosebush Gp=37	Q856Z7_9CAUD	2	0.35%	8.54%
Major tail protein Mycobacterium phage Rosebush Gp=21	Q857B3_9CAUD	3	0.53%	7.80%
Uncharacterized protein Mycobacterium phage Rosebush Gp=4	Q857D0_9CAUD	1	0.18%	6.99%
Uncharacterized protein Mycobacterium phage Rosebush Gp=31	Q857A3_9CAUD	2	0.35%	5.44%
Uncharacterized protein Mycobacterium phage Rosebush Gp=49	Q856Y5_9CAUD	2	0.35%	4.40%
Uncharacterized protein Mycobacterium phage Rosebush Gp=62	Q856X2_9CAUD	1	0.18%	3.43%
Uncharacterized protein Mycobacterium phage Rosebush Gp=45	Q856Y9_9CAUD	1	0.18%	3.15%
Uncharacterized protein Mycobacterium phage Rosebush Gp=38	Q856Z6_9CAUD	1	0.18%	2.75%
Bovine serum albumin precursor	CON__P02769	297	52.10%	0.00%
KRT1 Keratin, type II cytoskeletal 1	CON__P04264	57	10.00%	0.00%
TRYP_PIG Trypsin (Sus scrofa)	CON__P00761	37	6.49%	0.00%
ALB isoform 1 of serum albumin precursor	CON__P02768-1	35	6.14%	0.00%
KRT10 Keratin, type I cytoskeletal 10	CON__P13645	27	4.74%	0.00%
KRT9 Keratin, type I cytoskeletal 9	CON__P35527	24	4.21%	0.00%
KRT2 Keratin, type II cytoskeletal 2 epidermal	CON__P35908	11	1.93%	0.00%
Vitamin D-binding protein precursor (Bos taurus)	CON__Q3MHN5	9	1.58%	0.00%
54 kDa protein (Bos taurus)	CON__ENSEMBL:ENSBTAP00000018229	9	1.58%	0.00%
Krt10 isoform 1 of keratin, type I cytoskeletal 10	CON__P02535-1	8	1.40%	0.00%
SERPINA3-5 (Bos taurus)	CON__A2I7N1	7	1.23%	0.00%
KRT5 Keratin, type II cytoskeletal 5	CON__P13647	6	1.05%	0.00%
HRNR Hornerin	CON__Q86YZ3	6	1.05%	0.00%
SERPINA3-4 (Bos taurus)	CON__A2I7N0	5	0.88%	0.00%
Similar to endopin 2B (Bos taurus)	CON__REFSEQ:XP_001252647	5	0.88%	0.00%
KRT14 Keratin, type I cytoskeletal 14	CON__P02533	5	0.88%	0.00%
PRSS1 Trypsin-1 precursor	CON__P07477	5	0.88%	0.00%
Krt13 Isoform 1 of keratin, type I cytoskeletal 13	CON__P08730-1	5	0.88%	0.00%
Alpha-1-acid glycoprotein precursor (Bos taurus)	CON__Q3SZR3	4	0.70%	0.00%

Keratin, type I cuticular HA2	CON__Q14532	4	0.70%	0.00%
KRT16 Keratin, type I cytoskeletal 16	CON__P08779	4	0.70%	0.00%
Krt35 protein	CON__Q497I4	3	0.53%	0.00%
Similar to sex hormone-binding globulin (Bos taurus)	CON__Q6T181	3	0.53%	0.00%
KRT6A Keratin, type II cytoskeletal 6A	CON__P02538	3	0.53%	0.00%
KRT6C Keratin, type II cytoskeletal 6C	CON__P48668	2	0.35%	0.00%
Beta-casein (Bos taurus)	CON__P02666	2	0.35%	0.00%
Similar to carboxypeptidase N catalytic chain (Bos taurus)	CON__Q2KJ83	2	0.35%	0.00%
Coagulation factor V precursor (Bos taurus)	CON__Q28107	2	0.35%	0.00%
Similar to intersectin long isoform 4 (Bos taurus)	CON__ENSEMBL:ENSBTAP00000001528	2	0.35%	0.00%
9 kDa protein (Bos taurus)	CON__ENSEMBL:ENSBTAP00000038329	1	0.18%	0.00%
Tropomyosin 2 (Bos taurus)	CON__Q3SX28	1	0.18%	0.00%
44 kDa protein (Bos taurus)	CON__ENSEMBL:ENSBTAP00000024466	1	0.18%	0.00%
Alpha-1-antiproteinase precursor (Bos taurus)	CON__P34955	1	0.18%	0.00%
Keratin, type II cuticular Hb6	CON__O43790, CON__P78385, CON__Q14533,CON__Q6NT21	1	0.18%	0.00%
KRT73 Keratin-73	CON__Q32MB2	1	0.18%	0.00%
Apolipoprotein A-IV precursor (Bos taurus)	CON__Q32PJ2	1	0.18%	0.00%
Type I hair keratin KA35 (Homo sapiens)	CON__Q6IFU6	1	0.18%	0.00%

Table S4. Mass spectrometry data analysis of band 4 from SDS-PAGE of Rosebush capsid

Protein name	Protein accession numbers	Total spectrum count	Percentage of total spectra	Percentage sequence coverage
Uncharacterized protein Mycobacterium phage Rosebush Gp=17	Q857B7_9CAUD	101	21.80%	54.20%
Major tail protein Mycobacterium phage Rosebush Gp=21	Q857B3_9CAUD	7	1.51%	18.90%
Uncharacterized protein Mycobacterium phage Rosebush Gp=34	Q857A0_9CAUD	2	0.43%	17.10%
Uncharacterized protein Mycobacterium phage Rosebush Gp=37	Q856Z7_9CAUD	3	0.65%	15.10%
Uncharacterized protein Mycobacterium phage Rosebush Gp=53	Q856Y1_9CAUD	4	0.86%	14.70%
Uncharacterized protein Mycobacterium phage Rosebush Gp=5	Q857C9_9CAUD	1	0.22%	13.90%
Uncharacterized protein Mycobacterium phage Rosebush Gp=40	Q856Z4_9CAUD	1	0.22%	13.20%
Uncharacterized protein Mycobacterium phage Rosebush Gp=4	Q857D0_9CAUD	1	0.22%	6.99%
Uncharacterized protein Mycobacterium phage Rosebush Gp=58	Q856X6_9CAUD	1	0.22%	4.94%
Uncharacterized protein Mycobacterium phage Rosebush Gp=62	Q856X2_9CAUD	1	0.22%	3.43%
Uncharacterized protein Mycobacterium phage Rosebush Gp=45	Q856Y9_9CAUD	1	0.22%	3.15%
Uncharacterized protein Mycobacterium phage Rosebush Gp=42	Q856Z2_9CAUD	1	0.22%	2.60%

Uncharacterized protein Mycobacterium phage Rosebush Gp=49	Q856Y5_9CAUD	1	0.22%	2.20%
Uncharacterized protein Mycobacterium phage Rosebush Gp=15	Q857B9_9CAUD	1	0.22%	1.78%
Bovine serum albumin precursor	CON__P02769	143	30.90%	0.00%
TRYP_PIG Trypsin (Sus scrofa)	CON__P00761	53	11.40%	0.00%
KRT1 Keratin, type II cytoskeletal 1	CON__P04264	33	7.13%	0.00%
Alpha-1-acid glycoprotein precursor (Bos taurus)	CON__Q3SZR3	22	4.75%	0.00%
KRT10 Keratin, type I cytoskeletal 10	CON__P13645	19	4.10%	0.00%
ALB isoform 1 of serum albumin precursor	CON__P02768-1	17	3.67%	0.00%
KRT6C Keratin, type II cytoskeletal 6C	CON__P48668	14	3.02%	0.00%
KRT6A Keratin, type II cytoskeletal 6A	CON__P02538	13	2.81%	0.00%
KRT2 Keratin, type II cytoskeletal 2 epidermal	CON__P35908	11	2.38%	0.00%
KRT5 Keratin, type II cytoskeletal 5	CON__P13647	10	2.16%	0.00%
KRT9 Keratin, type I cytoskeletal 9	CON__P35527	9	1.94%	0.00%
KRT14 Keratin, type I cytoskeletal 14	CON__P02533	6	1.30%	0.00%
HRNR Hornerin	CON__Q86YZ3	5	1.08%	0.00%
Krt10 isoform 1 of keratin, type I cytoskeletal 10	CON__P02535-1	5	1.08%	0.00%
PRSS1 Trypsin-1 precursor	CON__P07477	4	0.86%	0.00%
FLG Filaggrin	CON__P20930	4	0.86%	0.00%
Coagulation factor V precursor (Bos taurus)	CON__Q28107	4	0.86%	0.00%
KRT23 Keratin, type I cytoskeletal 23	CON__Q9C075	3	0.65%	0.00%
Keratin, type II cuticular Hb6	CON__O43790, CON__P78385, CON__Q14533, CON__Q6NT21	3	0.65%	0.00%
KRT73 Keratin-73	CON__Q32MB2	3	0.65%	0.00%
KRT77 Keratin 77	CON__Q7Z794	3	0.65%	0.00%
Krt31 keratin complex 1, acidic, gene 1	CON__A2A5Y0	1	0.22%	0.00%
Similar to complement facor I (Bos taurus)	CON__Q32PI4	1	0.22%	0.00%
Fibrinogen alpha chain precursor (Bos taurus)	CON__P02672	1	0.22%	0.00%
IGHM protein (Bos taurus)	CON__Q1RMK2	1	0.22%	0.00%
Similar to alpha-2-macroglobulin isoform 1 (Bos taurus)	CON__ENSEMBL:ENSBTAP00000024146	1	0.22%	0.00%
Keratin, type I cuticular HA2	CON__Q14532	1	0.22%	0.00%
Similar to inter-alpha-trypsin inhibitor heavy chain 2 (Bos taurus)	CON__Q9TRI1	1	0.22%	0.00%
SERPINA3-5 (Bos taurus)	CON__A2I7N1	1	0.22%	0.00%
Beta-casein (Bos taurus)	CON__P02666	1	0.22%	0.00%
Similar to endopin 2B (Bos taurus)	CON__REFSEQ:XP_001252647	1	0.22%	0.00%
